# Effectiveness of photofunctionalized titanium alloy on osseointegration in rats with type 2 diabetes

**DOI:** 10.1186/s13018-022-03346-4

**Published:** 2022-10-08

**Authors:** Shengdao Jin, Yuji Yamamoto, Yoshifumi Harada, Sho Kaneko, Kazuki Oishi, Yasuyuki Ishibashi

**Affiliations:** Department of Orthopaedic Surgery, Hirosaki University Graduate School of Medicine, 5 Zaifu-cho, Hirosaki, Aomori 036-8562 Japan

**Keywords:** Ultraviolet, Ti6Al4V, Diabetes mellitus, Osseointegration, Photofunctionalization

## Abstract

**Background:**

Ultraviolet (UV) light-mediated photofunctionalization improves the osseointegration of pure titanium and titanium alloy (Ti6Al4V). However, little is known about the effect of UV irradiation on Ti6Al4V, used frequently in orthopedic surgery, in diabetic patients. We examined the effect of UV irradiation on Ti6Al4V in rats with type 2 diabetes.

**Methods:**

Cylinder Ti6Al4V implants were used. Half the animals were Sprague Dawley rats (the control group), and the other half were Spontaneously Diabetic Torii fatty rats (the diabetes mellitus model). For radiological analysis, bone density was observed and calculated using 3D microcomputed tomography. Histological analysis was performed to calculate the bone–implant contact (BIC) ratio. We used Pearson correlation to analyze the correlation between average blood glucose level and BIC ratio, and between average blood glucose level and bone volume (BV) ratio.

**Results:**

In the UV light-treated group, the BIC ratios of the normal and diabetic rats increased significantly compared with those in the untreated group at 2 weeks; at 4 weeks, the BIC ratio of the diabetic rats increased significantly, but there was no significant increase in the control animals. In both the control and diabetic groups, there was no significant difference in the BV ratios between the UV-treated and untreated implants at 2 or 4 weeks. The average blood glucose level in the 4-week group negatively correlated with the BIC and BV ratios. The average blood glucose level in the UV-treated group negatively correlated with the BIC ratio.

**Conclusion:**

Photofunctionalization of Ti6Al4V implants may promote osseointegration in the early stages in rats with type 2 diabetes.

## Background

As the world’s population ages, the number of total hip arthroplasties (THA) and total knee arthroplasties (TKA) are increasing rapidly worldwide [1–3]. Titanium alloy (Ti6Al4V) implants are often used as prosthetic devices and one of the main materials used for fracture fixation in orthopedic surgery because of their advantages, which include corrosion resistance, good biocompatibility, high stiffness, and a high weight-bearing tolerance [4–6]. Although there is a growing demand for such implant surgeries in an aging society, the number of patients with poor bone quality has increased, resulting in poor bone–implant integration, which often causes serious complications. Improving the speed and strength of bone–titanium integration remains a long-term challenge in the field of orthopedics [7, 8].

Diabetes mellitus (DM) is a common comorbidity associated with adverse outcomes in surgical patients. In 2019, the International Diabetes Federation announced that there were approximately 463 million diabetic patients worldwide, and this number is expected to reach 700.2 million by 2045 [9]. DM is a risk factor for poor osseointegration, surgical site infections, aseptic loosening, dislocation, periprosthetic fracture, readmission, and mortality after patients have undergone either THA or TKA [10–12]. Clinical studies demonstrate that patients with type 2 diabetes have higher implant failure rates than nondiabetic patients [13, 14], and diabetic animal models demonstrate significant reductions in osseointegration parameters, particularly in the percentage of bone–implant contact (BIC) [15–17].

Recently, it has been shown that irradiating implants with ultraviolet (UV) light improve associated cell growth and osseointegration [18, 19]. This innovative technology enhances osseointegration, and the BIC ratio of photofunctionalized titanium implants increased to a near-maximum level of 98.2% in a rat model, 1.9 times that of the untreated implants at 4 weeks. The effect of photofunctionalization on Ti6Al4V surfaces was demonstrated in vitro to enhance both bioactivity and osteoconductivity [20]. After UV irradiation, the carbon content of Ti6Al4V surfaces decreases significantly, and the hydrophilic surface becomes more hydrophilic (contact angles decreased from 72.3 to 6.0 degrees) [21]. Further, photofunctionalization is effective in improving the survival rate and stability of Ti6Al4V screws under loading conditions [22].

Photofunctionalization accelerated and enhanced levels of osseointegration, and overcame impaired osseointegration, on pure titanium in a rat model of type 2 diabetes [23]. However, the effect of photofunctionalized Ti6Al4V on in vivo bone histomorphometric parameters, such as the BIC ratio in diabetic patients, is still unclear. We aimed to examine the effect of photofunctionalized Ti6Al4V on osseointegration in rats with type 2 diabetes.

## Methods

Animal experiments were performed to demonstrate the effects of photofunctionalized Ti6Al4V on osseointegration in rats with type 2 diabetes. In this *in vivo* study, Ti6Al4V implants were inserted into rat femurs, the rats were killed after 2 or 4 weeks and radiological analysis was performed using microcomputed tomography, followed by histological analysis using undecalcified specimens. The study protocol (ethical code number: M19007) was approved by the Animal Research Committee of Hirosaki University, and all experiments were performed according to the Rules for Animal Experimentation of University.

### Photofunctionalization of Ti6Al4V implants

**Fig. 1:**
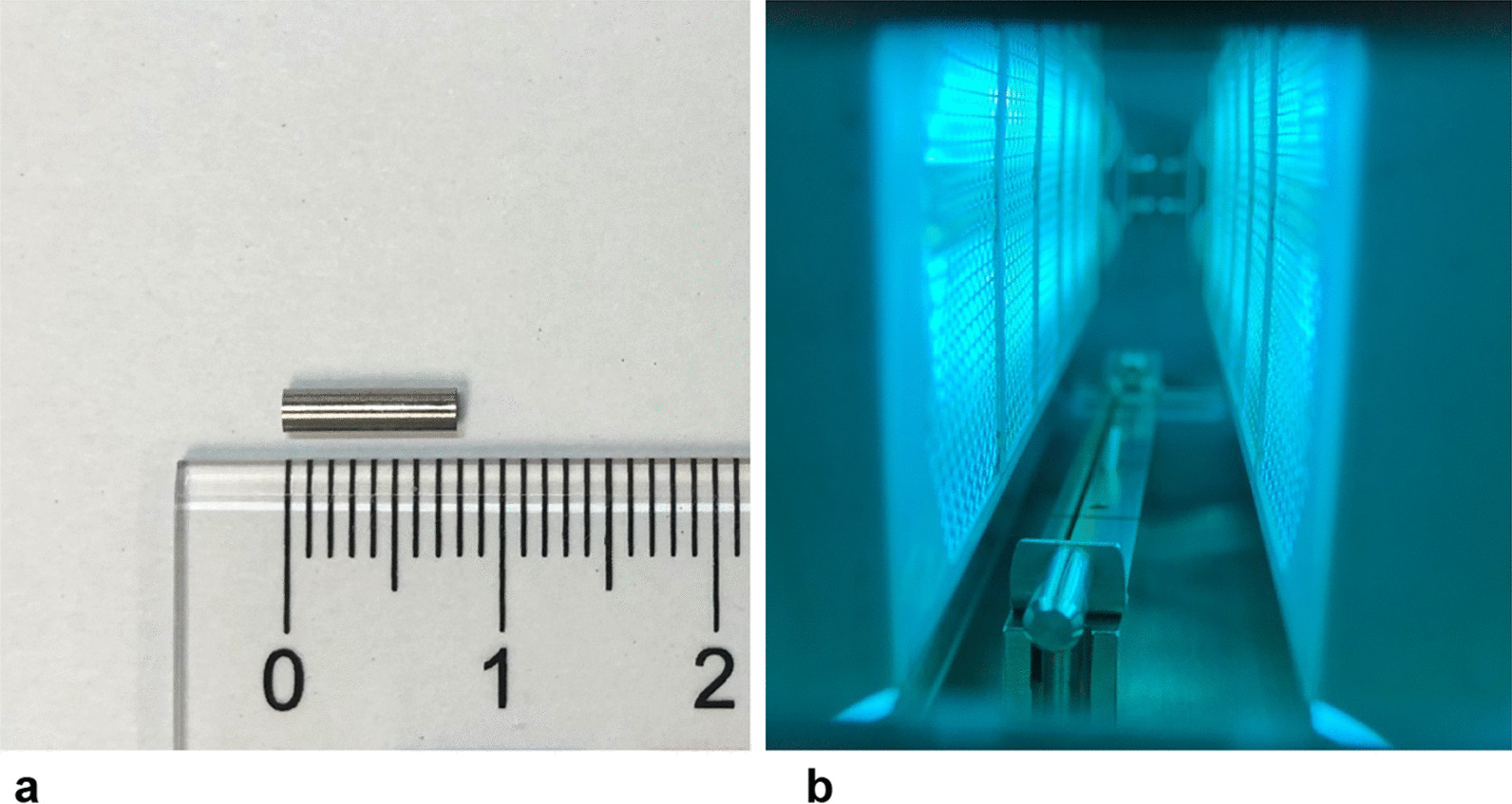
Photofunctionalization and implant. **a** Cylinder Ti6Al4V implants (diameter, 2 mm; length, 8 mm). **b** Implants were subjected to ultraviolet irradiation for 15 min

The cylinder implants were made from Ti6Al4V (diameter 2 mm, length 8 mm) and provided by B. Braun Aesculap Japan Co., Ltd (Tokyo, Japan). Half of the implants were treated with UV irradiation for 15 min using a photodevice (TheraBeam® affinity; Ushio Inc., Tokyo, Japan) at an intensity of 3 mW/cm^2^ (Fig. 1A–C). The light source mounted in the TheraBeam affinity was a low-pressure mercury (Hg) lamp, which emitted predominantly UV light of 185-nm and 254-nm. The implants were divided into two groups: Ti6Al4V without UV irradiation (untreated group) and Ti6Al4V with UV irradiation (UV-treated group).

### Animals

The Spontaneously Diabetic Torii (SDT) fatty rats (average adult weight of 360.7 ± 18.4 g) reach a high blood glucose level (approximately 200 mg/dL) at 6 weeks of age, and a very high level (approximately 400 mg/dL) at 8 weeks, and thus are a very mature model for type 2 diabetes [24–26].

Sprague Dawley (SD) rats (average adult weight of 266.7±13.5 g) were used for the control group and SDT fatty rats for the DM model. All rats were 8-week-old males and purchased from the same company (CLEA Japan, Inc., Tokyo, Japan). The rats were maintained in bracket cages and fed a standard laboratory diet and were able to access water *ad libitum* under temperature-, humidity-, and lighting-controlled conditions.

A total of 20 rats were divided into four groups, with five in each group as follows: Group I: SD rats implanted for 2 weeks; Group II: DM rats implanted for 2 weeks; Group III: SD rats implanted for 4 weeks; and Group IV: DM rats implanted for 4 weeks.

### Surgery

**Fig. 2:**
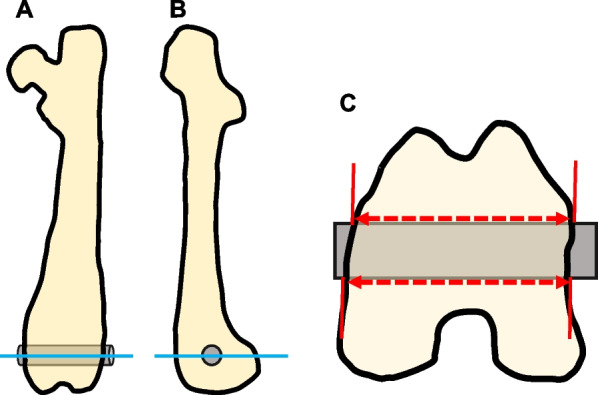
Illustration of the final position of the Ti6Al4V implant in the distal femur. **a** Anterior view of the left femur after implant (gray cylinder) placement. The blue line represents the surface through which the embedded specimen was cut using a microtome. **b** Lateral view of the left femur after implant placement. **c** Axial view of the cut surface in the distal femur. The measurement of the bone–implant contact (BIC) ratio was carried out at the area wherein the implant was inserted into the bone (range indicated by red arrow). Both the anterior and posterior surfaces of the bone tissue were analyzed

The rats were anesthetized with 1–2% isoflurane. Both hind limbs were shaved, and the incision area (from the distal femur to the knee) was wiped with alcohol before the skin and fascia were opened separately. The flat aspect of each distal femur was exposed and used for implantation. The bilateral distal femurs were drilled using a 2-mm diameter drill. UV-treated implants were inserted into the right femur holes, and untreated implants were inserted into the left femur holes (Fig. 2). After implant placement, the skin and fascia were closed with stitches. At either 2 or 4 weeks after surgery, the rats were euthanized by drawing more than 5 mL of blood directly from the heart, and the femurs were harvested.

### Blood glucose analysis

To confirm the establishment of normal and diabetic rats, blood glucose levels were measured just before implantation (0 weeks) and every 2 weeks after the operation until kill. Blood was collected from the tail of the rats, and blood glucose levels were measured using a blood glucometer (Experimental Animal Glucometer SUGL-001, ForaCare Japan, Japan).

### Radiological analysis

The specimens were analyzed using microcomputed tomography (Scan Xmate-L090, Comscantecno Co., Ltd., Japan). The imaging conditions were as follows: voltage, 80 kV; current, 100 μA; magnification, 4.942 times; resolution, 20.234 μm/pixel; and slice thickness, 20.234 μm. Three-dimensional bone morphometric analysis was performed using the TRI-3D-BON software (TRI/3D-BON, RATOC System Engineering Co., Ltd., Japan). The bone volume (BV) ratio was defined as the ratio of the mineralized BV within 100 μm from the implant surface. The BV ratio was calculated as the bone occupancy in the area of interest divided by the total area of interest, multiplied by 100.

### Histological analysis

The specimens were fixed in 10% buffered formalin and analyzed using microcomputed tomography (Scan Xmate-L090, Comscantecno Co., Ltd., Japan). Specimens were embedded in methyl methacrylate without decalcification [27]. The implants were left in situ for histological analysis. Embedded specimens were cut along the long axis of the implants using a microtome (EXAKT, Norderstedt, Germany).

Each 30–40 µm section was stained green with Villanueva–Goldner and examined by light microscopy (BZ-X700; Keyence Corp., Japan) to evaluate the bone area. For each histological slice, the BIC ratio for each group was calculated using digital image analysis software (ImageJ version 1.48). The BIC ratio was calculated as the length of the bone in direct contact with the surface of the implant divided by the total length of the implant, multiplied by 100 (Fig. 2). The bone in direct contact was defined as the interface at which the bone tissue was located within 20 μm of the implant surface without the intervention of soft tissue.

### Statistical analyses

The Mann–Whitney U test was performed to determine differences in blood glucose levels. The Wilcoxon signed-rank test was performed to determine differences in the BIC and BV ratios between the UV-treated and untreated groups at 2 or 4 weeks, respectively. The Mann–Whitney U test was also performed to determine differences in BIC and BV ratios between 2 and 4 weeks in each group. Statistical analyses were performed using SPSS (v 21.0; IBM), and *p*-values of < 0.05, were considered significant.

The Pearson correlation analysis method was used to analyze the correlation between mean blood glucose level and BIC ratio and between mean blood glucose level and BV ratio. Correlation analysis of all the data was performed, and the data were analyzed after different groupings. Correlation analyses were performed using SPSS (v 21.0; IBM), and *p*-values of < 0.05 were considered to indicate correlation. When *r* is > 0, the two variables are positively correlated, and when *r* < 0, the two variables are negatively correlated. When | *r* |≥ 0.8, the two variables were highly correlated; when 0.5 ≤| *r* |< 0.8, the correlation was moderate; when 0.3 ≤| *r* |< 0.5, there was a low correlation; and when | *r* |< 0.3, the correlation between the two variables was very weak and was regarded as uncorrelated.

## Results

### Blood glucose analysis

**Fig. 3:**
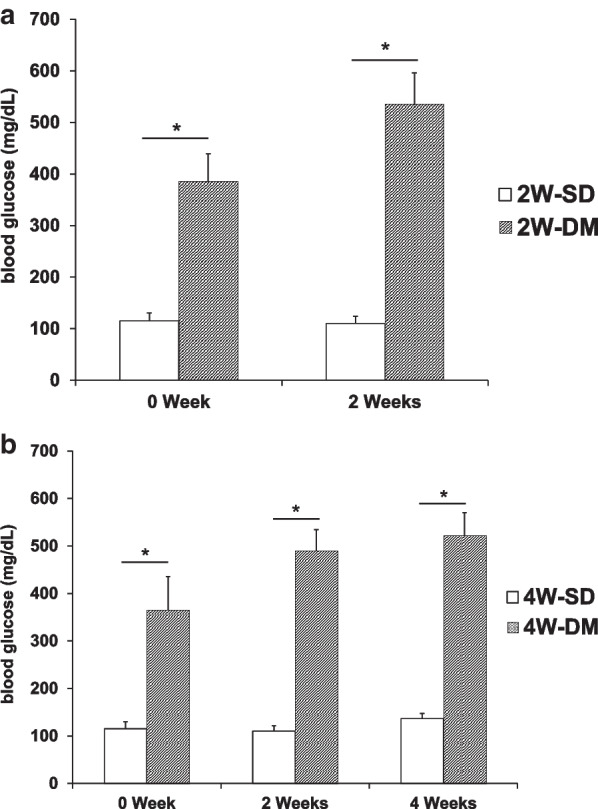
Comparison of the blood glucose level in normal (SD) vs. diabetic (DM) rats. **a** Mean blood glucose levels of the 2-week group in SD and DM rats. **b** Mean blood glucose levels of the 4-week group in SD and DM rats. Blood glucose levels were measured just before implantation (0 weeks) and every 2 weeks after the operation until kill. Results are presented as the mean percentage ± standard deviation. *p < 0.05 is considered as statistically significant between the normal and diabetic rats. SD: Sprague Dawley; DM: diabetes mellitus

Based on the blood glucose analysis, the blood glucose of Group I (SD rats implanted for 2 weeks) was determined to be 115.3 ± 15.0 mg/dL just before surgery (0 weeks) and 110.3 ± 14.9 mg/dL at 2 weeks after surgery (2 weeks) (Fig. 3a). The blood glucose of Group II (DM rats implanted for 2 weeks) was 384.4 ± 62.6 mg/dL (0 weeks) and 533.6 ± 71.1 mg/dL (2 weeks) (Fig. 3a). In the 4-week groups, the blood glucose of Group III (SD rats implanted for four weeks) was 115.2 ± 13.5 mg/dL (0 weeks), 110.2 ± 11.0 mg/dL (2 weeks), and 136.6 ± 10.6 mg/dL (4 weeks) after surgery (Fig. 3b). The blood glucose of Group IV (DM rats implanted for 4 weeks) was 364.4 ± 55.0 mg/dL (0 weeks), 489 ± 45.4 mg/dL (2 weeks), and 521.2 ± 49.0 mg/dL (4 weeks) (Fig. 3b).

At different time points, the blood glucose level of diabetic rats in each group was significantly higher (greater than 300 mg/dL) than that found in the healthy rats; thus, the diabetic model was established (Fig. 3a, b).

### Radiological analysis

**Fig. 4:**
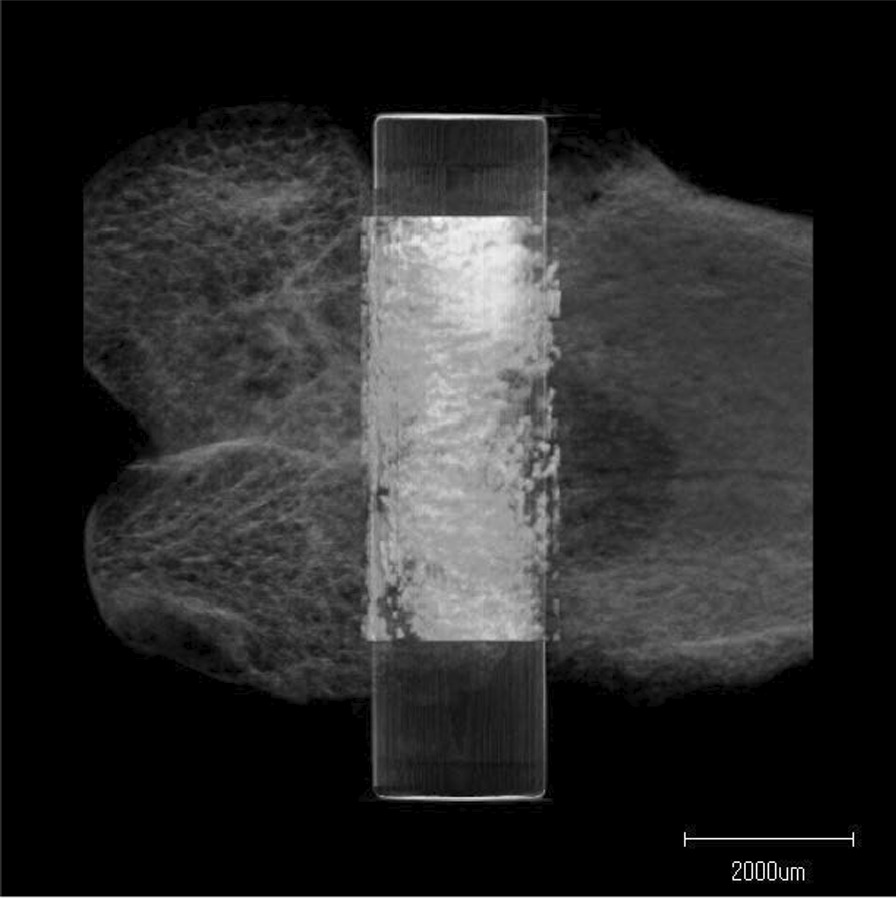
Microcomputed tomography (CT). Representative three-dimensional CT image around the implant is one of the samples from the diabetic 2-week UV-treated group

**Fig. 5:**
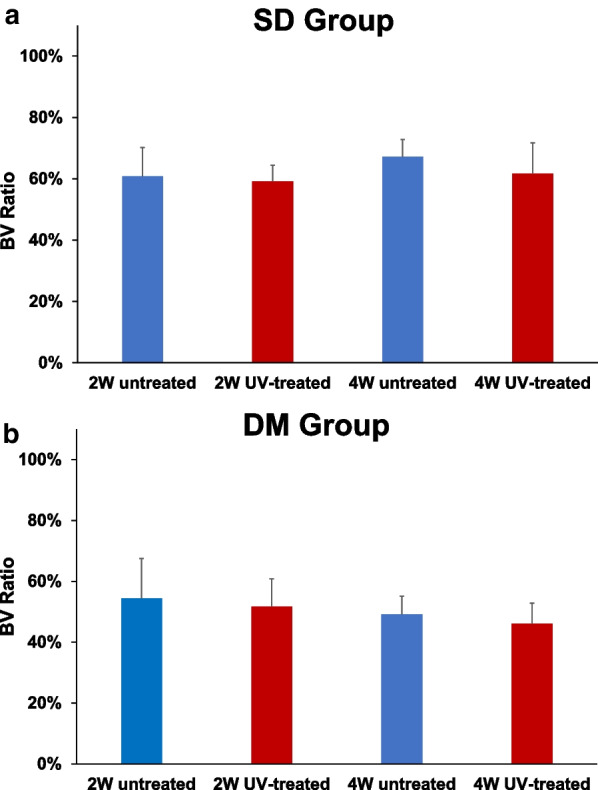
Bone volume (BV) ratio calculated using microcomputed tomography (CT). **a** Mean BV ratio of the normal rat (SD) group. **b** Mean BV ratio of the diabetic rat (DM) group. There were no significant differences in the BV ratio between untreated and UV-treated implants at 2 and 4 weeks in either the SD or DM group. 2 W: 2 weeks; 4 W: 4 weeks; SD: Sprague Dawley; DM: diabetes mellitus

In the SD groups, the mean BV ratio was 60.8% ± 9.3% for untreated rats and 59.2% ± 5.3% for UV-treated rats at 2 weeks; and 67.2% ± 5.6% for untreated rats and 61.7% ± 10.0% for UV-treated rats at 4 weeks (Figs. 4 and 5). There were no significant differences between the untreated and UV-treated groups at 2 or 4 weeks (*p* = 0.715 and *p* = 0.465, respectively).

In the DM group, the mean BV ratio was 54.4% ± 13.1% for untreated rats and 51.7% ± 9.1% for UV-treated rats at 2 weeks; and 49.2% ± 6.0% for untreated rats and 46.1% ± 6.8% for UV-treated rats at 4 weeks. Moreover, there were no significant differences between the untreated and UV-treated groups at 2 or 4 weeks (*p* = 0.686 and *p* = 0.068, respectively).

### Histological analysis

In the SD groups, the mean BIC ratio was 47.9% ± 8.2% for untreated rats and 67.0% ± 5.1% for UV-treated rats at 2 weeks; and 75.5% ± 12.9% for untreated rats and 79.9% ± 4.8% for UV-treated rats at 4 weeks. The BIC ratio increased significantly at 4 weeks compared with that at 2 weeks in both untreated and UV-treated groups (*p* = 0.016 and *p* = 0.008, respectively). There was a significant difference in the BIC ratio between the untreated and UV-treated groups at 2 weeks (p = 0.043); however, there was no significant difference at 4 weeks (*p* = 0.345).

**Fig. 6:**
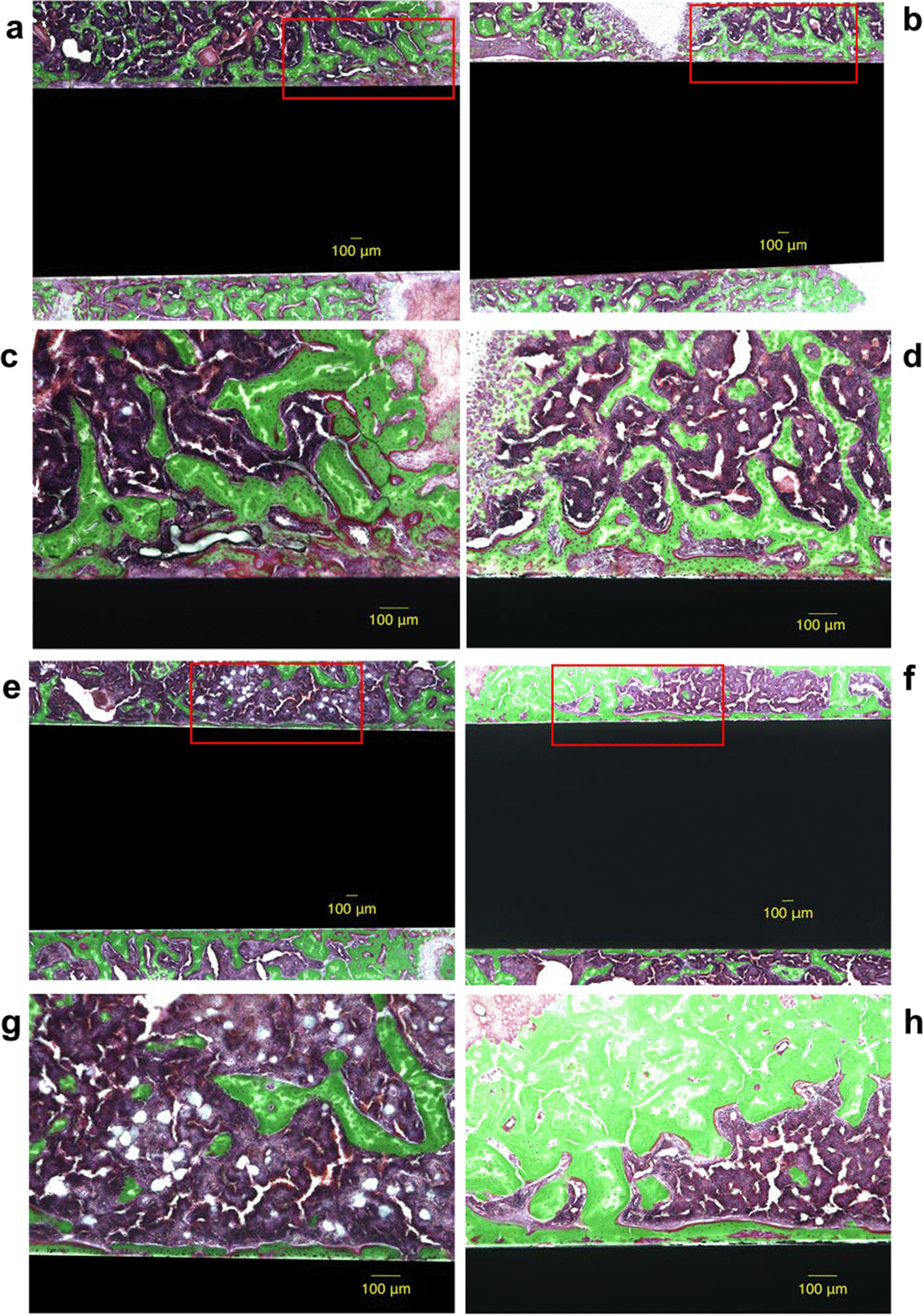
Light microscope images (magnification × 4 and × 10, higher-magnification images of the boxed areas). Light microscope images at 2 and 4 weeks after the implantation of the diabetes mellitus (DM) group. The micrographs show the bone response for the untreated (**a**, **c**, **e**, **g**) and ultraviolet (UV)-treated implants (**b**, **d**, **f**, **h**). A sample of the 2-week untreated group is shown in a and c, and sample from the 2-week UV-treated group is shown in b and d. One sample of the 4-week untreated group is shown in e and g, and a sample of the 4-week UV-treated group is shown in f and h. Scale bar: 100 μm

**Fig. 7:**
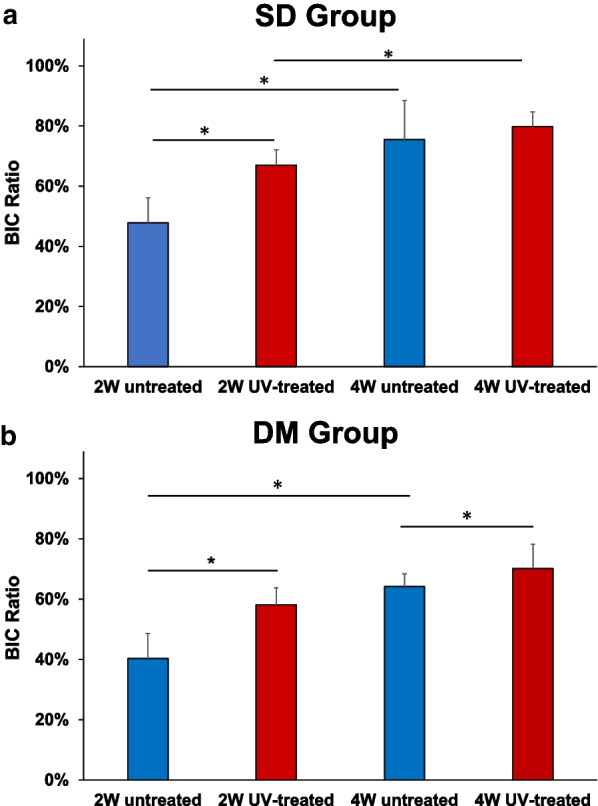
Histological analysis of bone–implant contact (BIC) ratio. **a** BIC ratios for untreated and ultraviolet (UV)-treated normal rat (SD) group are calculated. **b** BIC ratios for untreated and UV-treated diabetic rat (DM) group are calculated. Results are presented as the mean percentage ± standard deviation. *p < 0.05. SD: Sprague Dawley; DM: diabetes mellitus

In the DM group, the mean BIC ratio was 40.4% ± 8.3% for untreated rats and 58.1% ± 5.7% for UV-treated rats at 2 weeks and 64.2% ± 4.2% for untreated rats and 70.2% ± 8.0% for UV-treated rats at 4 weeks. The BIC ratio increased significantly at 4 weeks compared with that at 2 weeks in the untreated groups (*p* = 0.008); however, there was no difference in the UV-treated groups (*p* = 0.056). There were significant differences in the BIC ratio between the untreated and UV-treated groups at both 2 and 4 weeks (*p* = 0.043 and *p* = 0.043, respectively) (Figs. 6 and 7).

### Correlation analysis

Data analysis in all groups (Groups I, II, III, and IV) showed a slight negative correlation between mean blood glucose level and the BIC ratio (*r* =  − 0.368, *p* = 0.023); however, there was no correlation between mean blood glucose level and BV ratio (*r* =  − 0.220, *p* = 0.184). Data were divided into the 2-week (Groups I and II) and 4-week (Groups III and IV) groups for correlation analysis. The average blood glucose level in the 4-week group was moderately negatively correlated with the BIC ratio (*r* =  − 0.574, *p* = 0.008), and there was also a slight negative correlation between mean blood glucose level and BV ratio (*r* =  − 0.477, *p* = 0.034). Similarly, when data were divided into the UV-treated and untreated groups for analysis, the average blood glucose level in the UV-treated group was moderately negatively correlated with the BIC ratio (*r* =  − 0.586, *p* = 0.008); however, there was no correlation between mean blood glucose level and BV ratio (*r* =  − 0.302, *p* = 0.209).

## Discussion

We demonstrated that photofunctionalization can promote early phase osseointegration of Ti6Al4V in both type 2 diabetic rats and normal rats. The BIC ratios of surfaces at 2 and 4 weeks were significantly higher in the UV-treated group than in the untreated groups in diabetic rats. However, in normal rats, there was a significant difference between the UV-treated and untreated groups only at 2 weeks; there was no significant difference at 4 weeks.

Currently, limited research has been conducted on the photofunctionalization of Ti6Al4V implants in diabetic models. A previous study demonstrated that promoting osseointegration promoted bone integration of pure titanium in diabetic rats [23]. It reported that the integration of the UV-irradiated implants at 2 weeks was 80% higher than that of the control group [23]. Another clinical study showed that the healing time for implant stability in the moderately and poorly controlled diabetic groups was approximately twice as long as that in nondiabetic and well-controlled diabetes groups [28].

Recently, the biological aging phenomenon of titanium has been demonstrated to be a limitation. A reduced BIC ratio is reportedly caused by biological aging secondary to time-dependent biological degradation of the Ti surface [6]. Ti surfaces constantly absorb hydrocarbons from the atmosphere, together with water and cleaning solutions after the implants are manufactured [29]. The amount of carbon on the Ti surface affects the initial affinity of osteoblasts and amount of bone-Ti integration [6, 30, 31]. Additionally, the absorption of hydrocarbons leads to an increase in hydrophobia on the implant surface. Surface wettability is an important property for cell behavior, and by definition, cell attachment onto hydrophobic surfaces tends to be weaker than that onto hydrophilic surfaces [32–34]. Our previous study using the same implants as this study demonstrated that the amount of carbon and the contact angle on implants were significantly reduced after UV irradiation [21]. These changes of implant surface properties after UV irradiation enhance establishment of osseointegration in the early healing stage.

After UV irradiation, the osteoblasts on the surface of Ti6Al4V significantly increased by 80%–100% compared with those in the untreated group, thus enhancing the bioactivity and bone conductivity of Ti6Al4V [20]. UV irradiation can also enhance the osteogenesis around the implant, increase bone deposition on the titanium surface, and improve the sealing and support of the marginal bone [35]. Compared with the untreated implant, photofunctionalization of Ti6Al4V induced denser cortical bone formation and more rigid bone connection [23]. Similarly, this study demonstrated that photofunctionalization can promote early phase osseointegration of Ti6Al4V in both type 2 diabetic rats and normal rats. The fact that there was a significant difference in BIC between the UV-treated and the untreated groups and no significant difference in BV ratio was consistent with previous studies. While BIC evaluated new bone on the surface of the implant, BV ratio evaluated the proportion of bone tissue within 100 µm around the implant; it suggests that the photofunctionalization effect is strong near the surface of the implant.

Early osseointegration is essential because a low BIC rate in the early phase can very easily to lead to implant failure. Therefore, we evaluated the effect of photofunctionalization on Ti6Al4V surfaces in the early healing stage (up to 4 weeks). Increased hydrophillicity, which is one of the effects of UV irradiation, is reportedly observed at the implant surface after irradiation, but returns to its original state after 4 weeks [21]. Therefore, the effects of UV irradiation may not persist after 4 weeks. However, in this study, the BIC ratio of the diabetic rats in the UV-treated groups was approximately 10% lower than that in the control rats at 4 weeks. Further study is needed to definitively determine the lasting effects of photofunctionalization on Ti6Al4V in diabetics.

The correlations between mean blood glucose and BIC ratio and between mean blood glucose and BV ratio were also analyzed. There was no correlation between average blood glucose, BIC ratio, and BV ratio when data were analyzed, according to the 2-week and 4-week groups. However, the results of the 4-week group revealed a negative correlation between blood glucose and bone integration rate, and blood glucose and BV rate. If data were divided into UV-treated and untreated groups, the UV-treated group showed a negative correlation between blood glucose and BIC rate. Therefore, we believe that for a specific group, blood glucose will affect the BIC ratio or BV ratio, but further research is needed to confirm this.

This study had several limitations. First, there is no implant biomechanical test to evaluate the biomechanical strength of bone–implant integration, although studies have shown that the push-in values of photofunctionalized Ti and Ti6Al4V implants are significantly higher than those of control implants [18, 23]. Second, each group contained only five samples, making it difficult to draw statistical conclusions. Post hoc power analysis indicated that five specimens provided a power of 0.70 to detect the difference of BIC ratio between UV-treated and untreated groups (effect size = 1.5, *α* = 0.05). Third, to analyze bone regeneration around the implant, no fluorescence staining was performed. This method can be used as a dynamic evaluation method for bone regeneration.

## Conclusions

UV irradiation can promote the early osseointegration of Ti6Al4V in diabetic models. Blood glucose level, UV radiation, and the time after implantation are all important factors that affect osseointegration. The strict control of blood glucose levels in patients with diabetes will help bone–implant integration. UV irradiation can be applied to orthopedic implants for diabetic patients, to promote early load-bearing and potentially reduce the implant failure rate.

## Data Availability

The datasets used and analyzed during the current study are available from the corresponding author upon reasonable request.

## Abbreviations

UV: Ultraviolet
DM: Diabetes mellitus
Ti: Titanium
Ti6Al4V: Titanium alloy
BIC: Bone–implant contact
BV: Bone volume
TV: Tissue volume
